# Formulation, In Vitro and In Silico Evaluations of Anise (*Pimpinella anisum* L.) Essential Oil Emulgel with Improved Antimicrobial Effects

**DOI:** 10.3390/gels9020111

**Published:** 2023-01-28

**Authors:** Faizul Azam, Mohammed H. Alqarni, Sulaiman Mohammed Alnasser, Prawez Alam, Talha Jawaid, Mehnaz Kamal, Shamshir Khan, Aftab Alam

**Affiliations:** 1 Department of Pharmaceutical Chemistry and Pharmacognosy, Unaizah College of Pharmacy, Qassim University, Unaizah 51911, Saudi Arabia; 2 Department of Pharmacognosy, College of Pharmacy, Prince Sattam Bin Abdulaziz University, Al Kharj 11942, Saudi Arabia; 3 Department of Pharmacology and Toxicology, Unaizah College of Pharmacy, Qassim University, Unaizah 51911, Saudi Arabia; 4 Department of Pharmacology, College of Medicine, Imam Mohammad Ibn Saud Islamic University (IMSIU), Riyadh 13317, Saudi Arabia; 5 Department of Pharmaceutical Chemistry, College of Pharmacy, Prince Sattam bin Abdulaziz University, Al Kharj 11942, Saudi Arabia; 6 Department of Pharmacognosy & Pharmaceutical Chemistry, College of Dentistry and Pharmacy, Buraydah Private Colleges, Buraydah 51418, Saudi Arabia

**Keywords:** anise, essential oils, *Escherichia coli*, emulgels, microbial resistance, molecular docking

## Abstract

Over the past decade, researchers have made several efforts to develop gel-based formulations that provide an alternative to traditional hydrogels and emulgel. Due to its excellent antibacterial properties, anise, the main constituent of *Pimpinella anisum* L., widely used in pharmaceuticals, was selected as the active ingredient in this study. Since many bacteria have developed considerable antibiotic resistance, this research aimed to develop an herbal emulgel for treating skin infections caused by bacteria. Given these obstacles, we developed and evaluated a new, cost-effective topical emulgel solution containing anise essential oil against *Escherichia coli* (*E. coli*). Anise-based emulgels, potential drug delivery platforms, have been evaluated for various parameters, including physical properties, viscosity, pH, rheology, encapsulation efficiency, and in vitro release research. The AEOs emulgel demonstrated remarkable colloidal stability, with a zeta potential of 29 mV, a size of 149.05 nm, and considerable polydispersity. The efficacy of anise-loaded emulgels as antibacterial formulations was evaluated in vitro. *E. coli* was used as a model microbial organism for the antibacterial study. Human keratinocytes (HaCaT) were used to examine the biocompatibility of the emulgel. Molecular docking revealed that the essential oil components of *Pimpinella anisum* L. possess a high affinity for the bacterial adhesin protein FimH of *E. coli*. These findings indicate that the developed AEOs have the potential to be analyzed using *E. coli* as a model organism.

## 1. Introduction

Although antibiotics have been very successful in fighting infectious diseases, bacterial infections and drug resistance continue to be serious problems in healthcare worldwide [1,2]. An important factor contributing to the development of antibiotic-resistant bacteria is improper or excessive antibiotic use [3]. As microbial resistance increases, researchers are increasingly focusing on developing new antimicrobial chemicals and delivery technologies. As a serious health threat and economic liability, *Escherichia coli* (*E. coli*) causes enormous economic losses to humans and animals [4]. Bloody diarrhea, nausea, vomiting, dehydration, and abdominal discomfort can be symptoms of *E. coli* infections, which can even lead to septic shock and death [5]. It is an opportunistic pathogen that causes a wide variety of nosocomial infections once it forms a biofilm. These infections are often difficult to treat with currently available antibiotics [6].

Essential oils (EOs) obtained from plants consist of several volatile and non-volatile complex components. Chemically, they are rich in phenols, terpenes, and terpenoids, all of which have beneficial biological effects [7]. They are the essential constituents of aromatic and medicinal plants and mostly contain metabolites such as monoterpenes, sesquiterpenes, phenylpropenes, etc. [8]. EOs have been used for centuries as a means of treatment due to the unique bioactive chemicals they contain [9]. Thus, EOs have a number of beneficial medical properties, including antibacterial, antioxidant, anti-inflammatory, and antiviral activity [10]. In the United States, organically processed foods contain EOs that are classified as Generally Regarded as Safe (GRAS). Anise essential oils (AEO), due to their aromatic compounds such as terpenes, anisaldehyde, and estragol, are often used as flavorings and preservatives in a wide range of different industries, including pharmaceuticals, food, and beverages, and cosmetics [11]. Anise is utilized in conventional medicine to treat a variety of ailments owing to its antibacterial and antioxidant characteristics [12]

There has been a great deal of interest in essential oils as safe, natural antimicrobial agents, but little attention has been paid to them. Due to their low solubility in water, they are limited in how much they can be introduced into a system, thereby reducing their antimicrobial activity [13]. Strategies are therefore urgently needed to overcome these limitations. Encapsulation technology was developed as an efficient technique to improve the limitations of AEO, such as its poor bioavailability and unstable nature. This was done to address the difficulties of low bioavailability. One approach to overcome these limitations is nano-encapsulation of EOs in different delivery methods. The development of delivery technologies that improve drug solubility and bioavailability or distribute drugs locally to treat infections with fewer drugs and lower treatment doses is, therefore, highly desirable [3].

Despite the extensive research in *E. coli* treatment, the current treatment has attracted much attention. The formulation of drugs that are delivered via the skin varies from liquid to powder, but semisolid preparations are becoming more prevalent [14]. In this context, topical formulations such as emulgels have unique properties that can provide the benefits of emulsions and gels. They have a unique structure that allows for increased stability of active ingredients and can encapsulate both hydrophilic and lipophilic compounds [15]. Tirgarian and a team of researchers have successfully implemented the integration of emulsion gelation and nano-enhancement techniques to produce oleofilms with an exceptionally high concentration of vegetable oil. Moreover, this integration resulted in the oleofilms exhibiting remarkable structural stability [16].

Combining a nanoemulsion with a gel results in the formation of a semisolid product called a nanoemulgel, which has been shown to be an effective method of delivering drugs through the skin [17]. This is due in part to the ease of manufacture and low cost associated with the use of emulsifiers in the formulation of nanoemulgels [18]. The simple and inexpensive nature of the materials and methods used in the preparation of emulsifiers makes them a practical and cost-effective option for the preparation of nanoemulsions. Furthermore, the inclusion of a gel phase in the nanoemulgel formulation confers a number of desirable properties, such as a non-greasy texture, improved patient compliance, and enhanced cosmetic appeal. In addition, nanoemulgels have been found to possess a number of beneficial properties for dermatological use, such as rapid absorption, softness, and the ability to easily remove and spread evenly on the skin [19,20,21]. In addition, the improved external appearance, distribution and skin penetration of nanoemulgels enhance drug delivery [22].

Based on the available research, it is believed that the use of emulgel as a transdermal formulation holds significant potential for increasing the drug loading capacity while also improving the solubility and bioavailability of the active pharmaceutical ingredient. Furthermore, emulgel has been found to provide a safe and suitable environment for temperature-sensitive active pharmaceutical ingredients compared to other nanocarriers. The overarching objective of this study is to develop a novel emulgel-based transdermal formulation utilizing essential oil derived from *Pimpinella anisum* L. for treating *E. coli* infections, with the aim of providing a more effective method for managing deep skin bacterial infections. Through the application of molecular docking techniques, the binding interactions of the phytoconstituents present in the essential oil with the bacterial adhesin protein, FimH of *E. coli*, have been investigated to gain a deeper understanding of the mechanisms of action.

## 2. Results and Discussion

### 2.1. Physical Characterization

The colour, consistency, and homogeneity of the prepared transdermal emulgel-based formulation of AEOs are visually evaluated to observe the physical properties. The appearance of AEOs emulgel-prepared formulations ranged from off-white to cream-coloured, exhibiting excellent homogeneity and consistency. When applied to the skin, the prepared emulgel compositions did not produce any unpleasant odours and did not leave behind any oily residue. This is very visible. As a result, it was shown that the AEO emulgel formulation had one month of development, the AEOs emulgel-based transdermal formulation did not change in any way, and there was no noticeable difference between the formulation.

### 2.2. pH Evaluation

**Figure 1:**
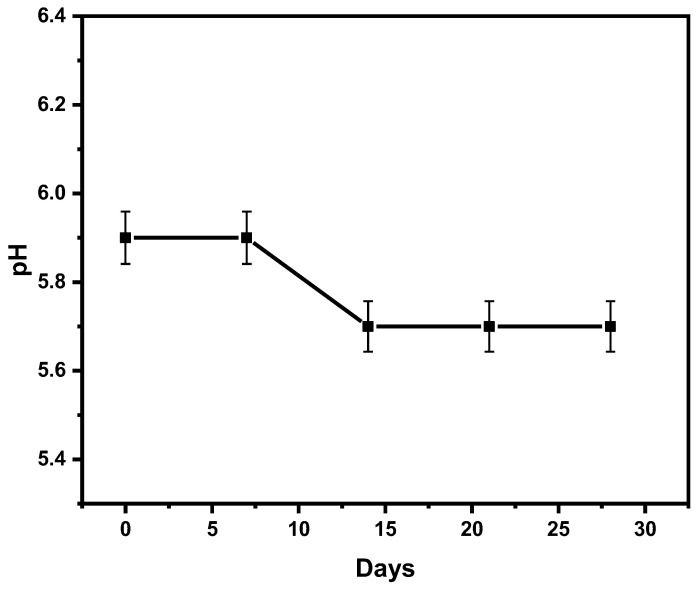
Evaluation of pH of AEOs emulgel-based transdermal formulation for 28 days.

It has been shown that the pH of the formulation is an extremely important parameter for its effectiveness and stability [23]. The pH of the formulation ranged from 5.9 ± 0.385 to 5.7 ± 0.25 from the 0th day to the 28th day. It was found that this was acceptable to avoid skin irritation, as shown in Figure 1. The pH of the transdermal formulation based on emulgel of AEO was reported to be 5.9 ± 0.385 at the beginning of the study, i.e., the first day is within the range of skin pH. No significant changes in the pH values of the formulations under storage conditions of 25 °C were observed. Still, a slight reduction in the pH value of the formulation was observed after 14 days. This decline in pH values over time could be due to the volatile nature of AEO.

### 2.3. Rheological Characterization

The rheological properties of a gel, such as viscosity and spreadability, are crucial in determining its performance and potential uses. Rheological behavior plays an important role in the assessment of topical preparations’ physical stability during storage and application. The viscosity of the prepared formula was observed for 28 days. The viscosity and spreadability of the formulation of AEO emulgel are shown in Table 1. The viscosity of emulgel compositions is often indicative of their consistency [24]. Topical applications require uniformity of the substance, since they are applied to thin layers of skin. The rheological properties of the gel play a significant role in controlling how much of the drug passes through the skin [25]. The synthesized AEO emulgel was found to have a viscosity of 5769 ± 288.45 cps on day zero. On the 7th day, 5765 ± 288.25 cps, and 5700 ± 260 cps on the 28th day. It was found that the emulgel formulation of AEOs is straightforward to disperse in a particular environment. It has been found that AEOs emulgel formulation has a spreadability of 6.17 ± 0.3085 cm. Gel treatment effectiveness is determined by its spread. Gels prepared for topical application must meet the optimal quality for topical application and have high spreadability. Additionally, this is considered essential for patient compliance with treatment [25]. Spreadability and viscosity study was aligned with the previous published paper of Snehal et al. [26].

### 2.4. Measurement of Particle Size, Polydispersity Index, and Zeta-Potential

**Figure 2:**
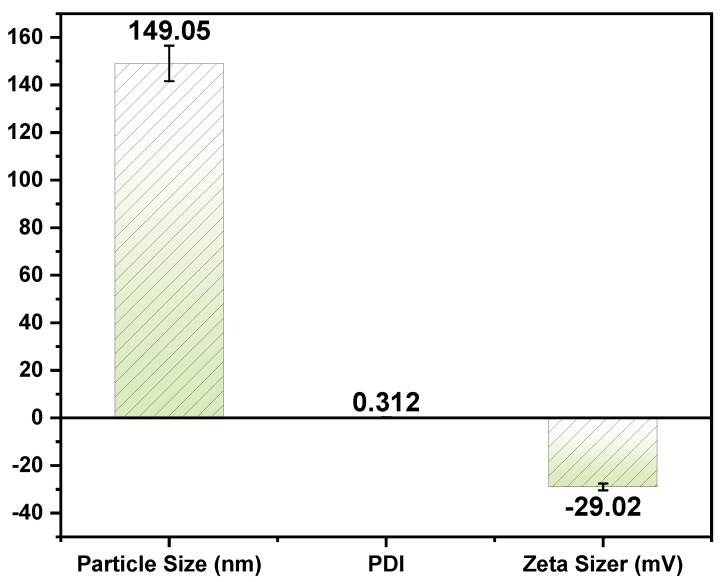
Measurement of particle size, polydispersity index, and zeta-potential AEOs emulgel-based transdermal formulation.

It is crucial to control the size of emulgels because they affect permeability, cellular absorption, half-life, and re-release of drugs [27]. Assessing the particle size of AEOs emulgel formulation was vital because it is a critical variable for the stability of the formulation. As indicated in Figure 2, the measured size of the particles was 149.05 ± 7.4525 nm. PDI stands for polydispersity index, which measures the distribution around the mean particle size. There is a range of values for PDI, ranging from 0 to 1, with higher values indicating a broader size distribution and possibly more aggregation within the sample. A substantially monodisperse sample is preferred in drug delivery systems to provide uniformity in drug release, cellular absorption, and % EE. A value of 0.3 or less is typically regarded as acceptable, whereas a value of 0.7 or over indicates that the particles have an exceptionally broad size range [28]. The PDI was reported to be 0.312 ± 0.0156. In order to determine the formulation’s physical stability, the zeta potential can be determined, which is a crucial characterization technique [29]. It was reported that the zeta potential of the AEOs emulsifier formulation was −29.02 mV, as shown in Figure 2. Higher ZP levels around 20 and 40 mV give stability to the systems and are less likely to cause an increase in particle size or the development of aggregates [30]. Colloidal stability can be predicted by the ZP size of NPs. NPs with ZP values that are either greater than or less than 25 mV tend to be highly stable [31]. Therefore, we concluded that our advanced formulation was highly stable. The ZP plays a significant role in determining the stability of colloidal suspensions and the behavior of particles in solutions.

### 2.5. Morphology

**Figure 3:**
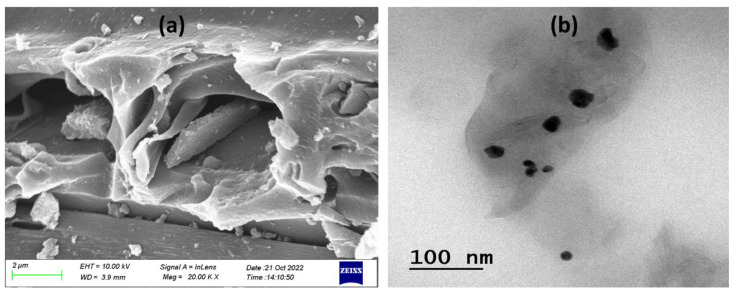
Morphology study (**a**) SEM and TEM (**b**) image of AEOs emulgel. 0.001 wt.% emulgels were dried at 24.5 °C in both cases.

Characterizing the composition, interfacial strain and structure, morphology, phase quantification, and size distribution of nanomaterials is a critical step in the design process. Both scanning and transmission electron microscopy (SEM/TEM) are widely used for this purpose [32]. SEM is one of the most famous experimental techniques for investigating and evaluating the imaging and characterization of micro- and nanoparticles. The resolution of 10 nm, or 100 Å, is one of the reasons why SEM is recommended for particle size investigation [33]. The structural morphology was found to be irregular and heterogeneous using SEM, as seen in Figure 3a. The morphology of the AEOs emulgel was later observed under TEM. In this case, a spherical morphology in shape has been observed, as shown in Figure 3b. Furthermore, TEM observations allow the differentiation of smaller and larger particles [34]. Finally, TEM images confirmed a population of monodispersed sphere drops with a smooth surface.

### 2.6. % Entrapment Efficiency (%EE)

The encapsulation efficiency of AEOs in emulgel has been investigated as a function of the essential oil initial amount. The % EE was reported to be 58.42 ± 7.35%. These results propose that a relatively high amount of AEO was entrapped emulgel.

### 2.7. Ex-Vivo Retention Studies

**Figure 4:**
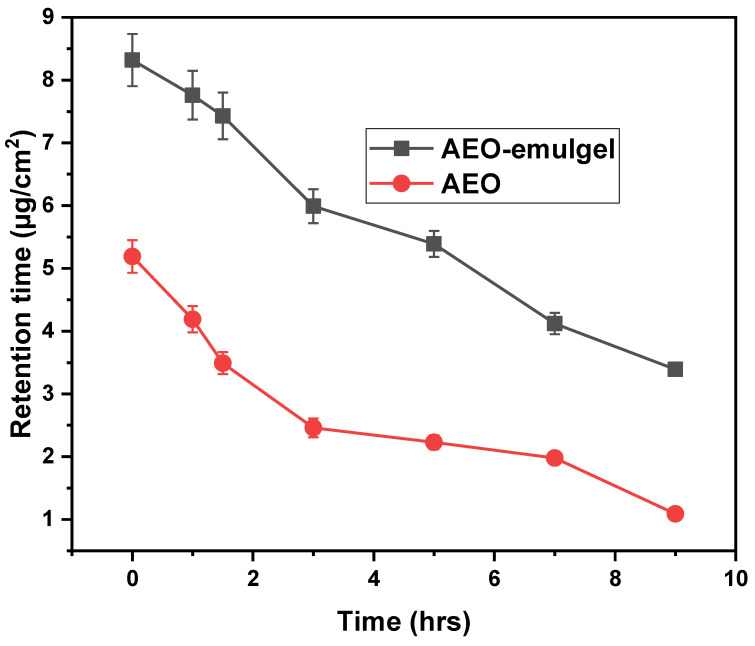
Ex-vivo retention studies of AEOs emulgel for 9 h.

Studies on the retention of drugs in the skin are essential components of local infection and targeting. The accumulation of drugs in specific tissues is an essential component in the design of effective drug carrier systems [35]. Ex vivo retention experiments were then performed to determine the amount of AEOs emulgel retained in the skin of mice at a pH of 6.0 for varying lengths of time, as shown in Figure 4. Initially, drug retention was reported to be 8.32 ± 0.416 μg AEOs emulgel, whereas in the case of AEO, 5.19 ± 0.259 μg. It was found that after 3 h the drug retention for AEOs emulgel was found to be 5.99 ± 0.269 μg and in the case of AEO, 2.46 ± 0.149 μg. After 9 h, the drug retention for AEOs emulgel was reported to be 3.39 ± 0.103 μg. The drug retention in AEO was reported to be 1.09 ± 0.054 μg. The result suggests that retention was high in the case of AEO’s emulgel compared to AEO. Thus, it was found that the amount of drug remaining in the skin was higher in the case of AEOs emulgel than AEO alone. The study found that the AEOs emulgel formulation had improved drug retention, which was due to the penetration-enhancing effect of the gel, which reduced water loss from the skin, resulting in hydration that led to improved retention of the oil in the skin. This was due to the fact that the gel increased the amount of drug that could be absorbed by the skin [36]. In the case of emulgel formulations of AEOs, this dermatological preservation of AEOs has been associated with the increased contact with the skin that these formulations create. Due to their presence in the emulgel formulation, essential oils, also called AEOs, contribute to the penetration and retention of AEOs in the skin by reversibly altering the complex structure of the skin. It was found that emulgel formulations were primarily responsible for the increased retention of AEOs in the skin, leading to this conclusion.

### 2.8. In-Vitro Drug Release Studies

**Figure 5:**
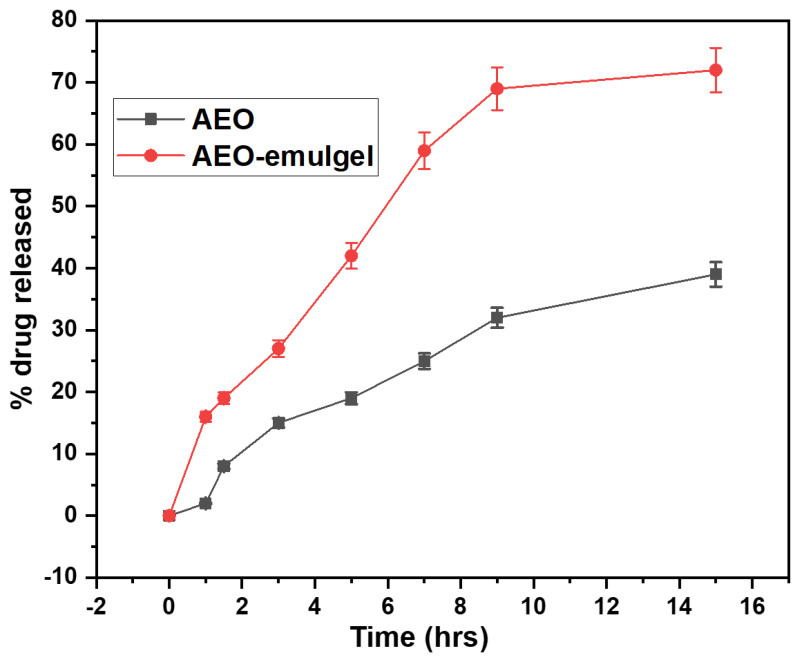
Comparative in-vitro drug released study of AEO and AEO-emulgel at pH 6 for 15 h.

In vitro drug release studies were performed using a dialysis tube. The amount of drug released was plotted against time, as shown in Figure 5. Initially, there was an abrupt release of AEO followed by a sustained release behaviour. The active ingredient is on the surface of emulgels and is not correctly incorporated into emulgels, resulting in an initial erratic release of AEO. The percent cumulative drug release of AEOs emulgel was 16 ± 0.8%, while the bare AEOs was 2 ± 0.1% in the first 1 h. After 5 h, the drug release from AEOs emulgel was 42 ± 2.1%, while the drug release from bare AEOs was 19 ± 0.95%. After 15 h, the release of the drug from the AEO emulgel was 72 ± 3.6%, while the release of the drug from the bare AEO was 39 ± 1.19%. It was found that the lowest percentage of the drug was released from the bare AEO compared to the AEOs emulgel. These data suggest that the carbopol gel contributes to drug release, and the emulgel with AEOs in the formulation plays a vital role in drug release from the gel over several hours. The Carbopol gel acts as a reservoir into which the drug penetrates and treats the targeted area of infection.

### 2.9. In-Vitro Cell Viability Assay

**Figure 6:**
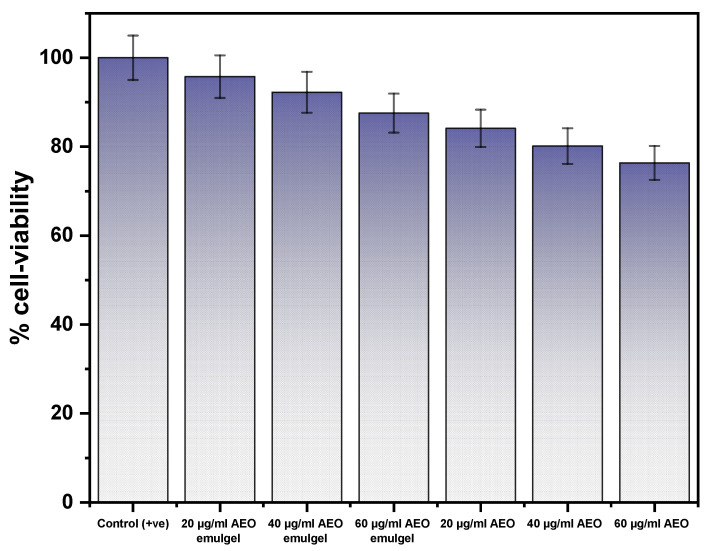
Comparative cell-viability assay for AEOs and AEO emulgel at a concentration ranging from 20 µg/mL–60 µg/mL in HaCaT.

The in vitro cell viability assay was performed on the human keratinocyte cell line (HaCaT) for AEOs and AEO emulgel at a concentration ranging from 20 µg/mL–60 µg/mL, as shown in Figure 6. The graph shows that the cells were more than 75% viable in both cases, i.e., bare AEOs and the AEO emulgel. In case of AEO-emulgel at the concentration of 20 µg/mL, the viability was observed to be 95.762 ± 4.78%, whereas in the case of AEOs, the viability was observed to be 84.121 ± 4.20%. With increasing concentration up to 60 µg/mL, the viability was decreased in both cases, i.e., 87.538 ± 4.3% (AEO-emulgel) and 76.34 ± 3.81% (bare AEOs). Whereas 100% cell was viable in incase of control HaCaT. These results showed that, interestingly, AEOs and AEO emulgel did not induce cell shrinkage, necrosis, or apoptosis, at least at concentrations below 60 µg/mL. Therefore, the developed formulation is safe and stable for further use.

### 2.10. Minimum Inhibitory Concentration

The term “minimum inhibitory concentration” (MIC) refers to the lowest concentration of an antimicrobial drug that prevents the observable growth of bacteria during an overnight incubation period [37]. As shown in Table 2, the most abundant compounds in all samples were AEO emulgels. The MIC was reported to be 1.02 µg/mL, while the MIC in the case of AEO emulgel was 4.7 µg/mL. Therefore, it can be concluded that the MIC value in the case of AEO emulgel treatment can significantly improve the efficacy of AEO-based therapy.

### 2.11. Molecular Docking

**Figure 7:**
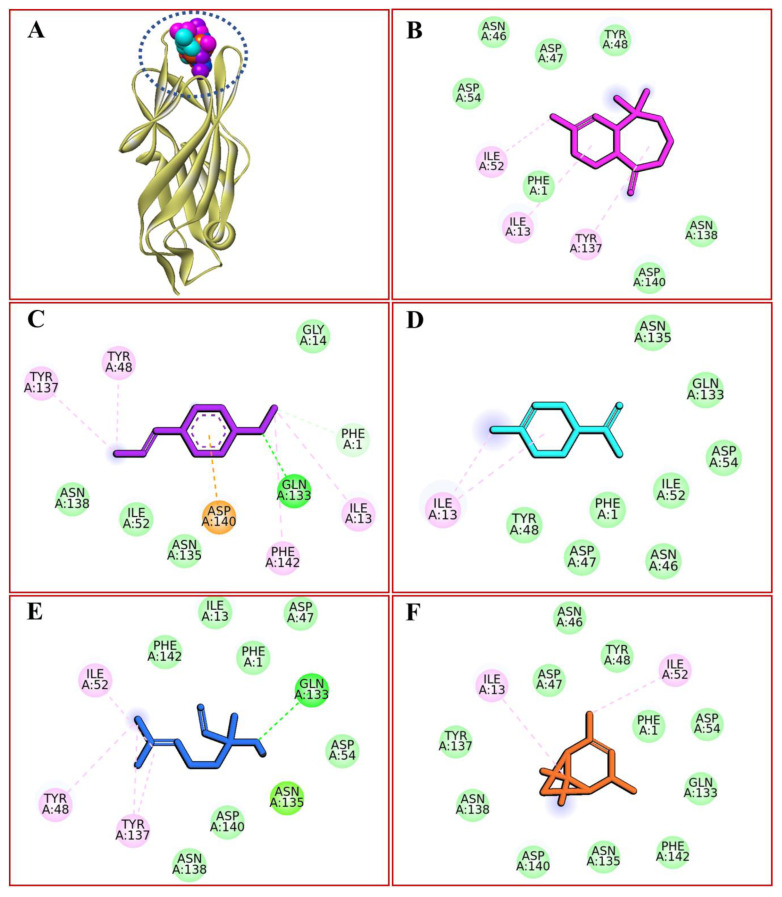
Intermolecular interactions of *Pimpinella anisum* L. essential oil components (shown as CPK style) with adhesin protein FimH of *E. coli*, depicted as ribbon (**A**); α-himachalene, shown as stick in pink colour (**B**); anethole, shown as stick in purple color (**C**); limonene, shown as stick in cyan color (**D**); linalool, shown as stick in blue color (**E**); *trans*-verbenol, shown as stick in brown color (**F**).

Using a molecular docking strategy, *Pimpinella anisum* L. essential oil components were tested for their ability to bind to the adhesin protein FimH of *E. coli*. Docking predicted binding energies revealed that α-himachalene possessed the highest affinity for the bacterial adhesin FimH protein, with a binding energy of −6.2 kcal/mol. In contrast, limonene was revealed to be the least active compound, with a value of −4.6 kcal/mol. On the other hand, trans-verbenol and linalool demonstrated moderate affinity showing −5.8 and −5.1 kcal/mol, respectively. Figure 7 depicts that docked compounds were engaged in hydrophilic and hydrophobic molecular interactions. Common residues contributing hydrophobic interactions included Ile13, Tyr48, Ile52, Tyr137, Asp140, and Phe142, whereas Gln133 afforded a hydrogen bond with anethole and linalool. However, Asp140 mediated a hydrogen bond with trans-verbenol.

## 3. Conclusions

In recent years, there has been a growing interest in developing gel-based formulations as an alternative to traditional hydrogels. This is due to the increasing resistance of bacteria to antibiotics and the desire for natural and cost-effective solutions. The main active ingredient in this study is anise, an herb commonly used in pharmaceuticals and known for its excellent antibacterial properties. The research aimed to develop a herbal emulgel for treating skin infections caused by bacteria, using anise essential oil (AEO) as the active ingredient. The emulgel was evaluated for various parameters such as physical properties, viscosity, pH, rheology, encapsulation efficiency, and in vitro release. The results showed that the AEO emulgel had remarkable colloidal stability and high encapsulation efficiency. In addition, the emulgel was effective against *Escherichia coli* (*E.coli*) in vitro, making it a potential treatment for bacterial skin infections. The emulgel was also biocompatible with human keratinocytes (HaCaT), which is a good indication of its safety for topical use. Molecular docking studies revealed that the essential oil components of *Pimpinella anisum* L. have a high affinity for the bacterial adhesin protein FimH of *E. coli*, providing insights into how the emulgel may work to treat bacterial infections. Compared to the effects of pure oil, the emulgel had much stronger antibacterial and anticancer properties due to its small particle size and large surface area, improving the interaction between the emulgel and bacteria. This study presents a new, cost-effective topical emulgel solution containing AEOs as a potential alternative for treating skin infections caused by bacteria. The results pave the way for further research to fully understand the mechanisms of action of AEOs in treating bacterial infections, focusing on the bacterial adhesin protein FimH of *E. coli*.

## 4. Materials and Methods

Anise oil, Carbopol 940, span 80, tween 80, paraffin liquid, and triethanolamine were purchased from Sigma-Aldrich, USA, and Merck. All the reagents used in the study were analytically graded. *Escherichia coli* (ATCC 11229) was collected from the Department of Pharmaceutics, College of Pharmacy, Prince Sattam Bin Abdulaziz University, Al-Kharj. Solvents and other chemicals were also purchased from Sigma Merck. Nutrient media and Sabouraud dextrose were purchased from HiMedia (Mumbai, India). Anise oil is composed primarily of anethole, which accounts for 80–90% of its chemical composition. Other compounds present in lesser concentrations include estragole, p-anisaldehyde, anise alcohol, acetophenone, pinene, and limonene [38].

### 4.1. Preparation of AEO-Nanoemulsion

A thorough methodology was used to prepare the AEO emulgel, which involved the creation of an emulsion through the careful and deliberate combination of an oil and aqueous phase. Using the procedures described by Burki et al., 2020 and Shakee et al., 2022 [18,36], the oil phase was first prepared by mixing 5 mL of paraffin liquid with 0.5 mL of Span 80 in a vortex mixer, ensuring constant mixing throughout the process. This mixture was then set aside for later use. The aqueous phase was then prepared by mixing 0.4 mL of Tween 80 with 5 mL of distilled water using a vortex mixer. These two phases were then combined and mixed for 20 min using the vortex mixer. To achieve a final concentration of 2%, the required amount of AEO was added to the nanoemulsion solution. The mixture was then homogenized at 15,000 rpm for 20 min, using an ice bath to prevent the evaporation of active volatile compounds and to withstand the heat generated by the rapid shaking. The container was also covered with aluminium foil to protect it from further evaporation [39].

### 4.2. Preparation of AEO Emulgel

In preparing an emulgel loaded with AEO, Carbopol 940 was used as the primary thickener at a concentration of 1%. In a beaker, 100 mL of distilled water and 1 gm of Carbopol 940 were mixed and continuously homogenized to obtain a dispersion. Triethanolamine was added dropwise to the dispersion mixture to obtain a transparent gel composition. The pH was then precisely adjusted with a few drops of triethanolamine, which finally resulted in the formation of a gel [39]. Next, the carefully prepared AEO emulgel was seamlessly integrated into a Carbopol 940 dispersion. The mixture was shaken vigorously for 15 min and stored at room temperature for 24 h.

### 4.3. Characterization of AEO Emulgel

#### 4.3.1. Physical Characterization

The prepared emulgel was inspected visually for colour, homogeneity, consistency, and phase separation.

pH evaluation: The pH of emulgels was measured using a pH meter. The pH meter was calibrated before use to ensure accurate results. A small amount of the emulgel sample was placed on the pH electrode, and the pH value was recorded. The pH of the emulgel was measured at room temperature, i.e., 25 °C and under controlled conditions to ensure the reproducibility of results. Average values were calculated after the test was carried out in triplicate.

#### 4.3.2. Rheological Characterization

Viscosity: The viscosity of the emulgel was determined by a Brookfield viscometer using a spindle. After four weeks of emulgel formulation, the viscosity was measured by gradually increasing the spindle’s rotational speed (10 to 30 rpm) and then gradually reducing it (10 to 30 rpm).

Spreadability: The 0.5 g gel was weighed and placed between two (20 × 20 cm) horizontal plates. The upper plate was loaded with 500 g of weight and left for around 5 min. The dispersion circle’s diameter is expressed in centimetres (cm). The outcomes are the average of three assessments.

#### 4.3.3. Measurement of Particle Size, PDI, and Zeta-Potential

After the preparation of the nanoemulgel, the Zetasizer Nano ZS90 (Malvern Instruments Ltd., Malvern, UK) at 25 °C was used to measure the emulgel’s particle size, PDI, and zeta potential. To eliminate the impact of pH and ionic strength on the zeta potential measurement during dilution, suitable emulsifier solutions were chosen per the equipment guidebook.

#### 4.3.4. Morphology

The morphology or shape of the AEO emulgel was examined using a field emission scanning electron microscope (FESEM; Philips XL 30 microscope, Hillsboro, OR, USA). The sample was mounted to a copper stub using double-sided adhesive tape. After that, the sample was evaluated in the FESEM at an excitation voltage of 5 kV after sputtering with gold at 20 mA for 120 s [40]. After this, Transmission electron microscopy (TEM, JEM-1010, JEOL Ltd., Tokyo, Japan) was performed. The AEO emulgel sample for the TEM analysis was prepared by the sonication method [41].

### 4.4. % Entrapment Efficiency (%EE)

The encapsulation efficiency (%EE) of essential oils (EOs) was quantitatively determined using a previously established method with slight modifications [42]. Specifically, 100 mg of the AEO emulgel was added to 5 mL of a 1-molar hydrochloric acid solution and heated in a boiling water bath for 30 min. After cooling, 1 mL of ethanol was added, and the mixture was centrifuged at 20,000 rpm for 1 min at a temperature of 4 degrees Celsius. The supernatant solution and AEO emulgel were then separated, and the concentration of AEO in absolute alcohol was determined spectrophotometrically at a wavelength of 298 nm. For comparison, a control or blank sample was prepared by replacing the sample dispersion with an appropriate amount of water. The following formula was used to calculate the entrapment effectiveness and loading capacity of AEOs:

(1) $$\%\;\mathrm{EE}=\frac{\left(Total\;amount\;of\;\mathrm{AEOs}\;added-Amount\;of\;free\;\mathrm{AEOs}\;in\;supernatent\right)}{Total\;amount\;of\;\mathrm{AEOs}\;added}\times 100$$

### 4.5. Ex-Vivo Retention Studies

Franz diffusion cells on mouse skin samples were used to study ex vivo skin retention of AEO-loaded emulgels and AEOs. Time-dependent skin samples were collected and thoroughly cleansed three times with 5 mL of normal saline. Samples were then minced and homogenized with distilled water for 10 min. The homogenates were centrifuged at 10,000 rpm for 15 min, and the resulting supernatant was filtered through a membrane with a pore size of 0.45 micrometres [36].

### 4.6. Drug Release Studies

The dialysis bag method is commonly used for evaluating drug release in an in vitro setting. The method involves placing AEO emulgel in a dialysis bag immersed in 500 mL of pH 6 PBS (phosphate buffered saline) at 50 rpm rotation speed. The dialysis bag is made of a semi-permeable membrane that allows small molecules such as drugs to diffuse out of the bag, while larger molecules such as proteins are retained within the bag. The pH 6 PBS solution simulates the physiological environment and allows for the assessment of the drug’s release rate. The drug release rate is determined by measuring the drug concentration in the PBS solution at various time intervals and determined by spectrophotometry at 298 nm.

### 4.7. In-Vitro Cell Viability Assay

MTT assay for cell viability was achieved using AEO emulgel in HaCaT cells using the following technique [1,43]. The HaCaT cells were taken from King Saud University in Saudi Arabia. These cells were then cultured using a modified version of Dulbecco Eagle medium (DMEM) which was supplemented with 10% (*v*/*v*) heat-activated fetal bovine serum. The cells were kept in a humidified incubator at a temperature of 37 °C with a CO_2_ concentration of 5%. Once the cells reached 80% confluence, they were split (1:4) and harvested using the trypsinization (1:4) method. The growth media was replaced every three days to ensure optimal growth and development of the cells.

### 4.8. Minimum Inhibitory Concentration

#### 4.8.1. Preparation of Inoculum

MIC was performed in sterile microtiter plates. Briefly, a stock solution of bare emulgel and AEO emulgel was prepared in water to ensure complete solubilization at 1 mg/mL concentration. A total of 100 µL of nutrient broth and Sabouraud dextrose broth were added to wells 1 to 10 bare emulgel and AEO emulgel samples (100 µL) were added to the first well. Serial dilution of the solution was performed from well 1 to well 10, and 100 µL from well 10 was discarded. All of the dilution wells from well 1 to well 10 received 100 µL of the bacterial suspension. In well 11, 100 µL of the overnight bacterial suspension (*E. coli*-ATCC 11229) was added, and 100 µL of sterile broth to act as a positive control or growth control. In well 12, 200 µL of sterile nutritional broth and Sabouraud dextrose broth were used as negative or sterility control. Then, the plate was incubated for 24 h at 37 °C. After incubation, utilizing an ELISA reader (Erba) with a 640 nm wavelength, the absorbance of each well was determined. For each microbial strain, the above-described process was used. The sample and standard concentrations that inhibit 50% of bacterial growth were determined for all microorganisms. To reduce error, every test was carried out in triplicate.

#### 4.8.2. Molecular Docking

The affinity of *Pimpinella anisum* L. phytoconstituents with the bacterial adhesin protein FimH of *E. coli* was calculated using molecular docking. From the RCSB PDB database (https://www.rcsb.org/ accessed on 30 December 2022), the 3D structure of the bacterial adhesin protein FimH from *E. coli* with the PDB ID of 4XO8 was retrieved. The PubChem database was used to collect the 3D structures of the chemical constituents of *Pimpinella anisum* L. Through the use of the Biovia Discovery Studio Visualizer 2021 program, the molecules were converted to PDB format. The software M.G.L. Tools (version 1.5.7) was utilized to add hydrogens, combine polar hydrogens, and calculate the charges of the ligand and protein receptors. In addition to analyzing the torsion root of the ligands, M.G.L. Tools was also used to identify the docking site in the protein. Then, the format of the protein and ligand was converted to pdbqt. Molecular docking was performed using AutoDock Vina 1.1.2 with an exhaustiveness value of 100 and all other parameters left at their default values [44]. The grid box was centered at −20.294, −6.717, and −16.395 in the x, y, and z directions, maintaining a size of 24 in all three dimensions. Using the Biovia Discovery Studio Visualizer 2021 software, the conformations of the docked ligands within the target protein were visualized after the docking experiment was completed, as previously discussed [45].

## Figures and Tables

**Table 1:** Viscosity and spreadability of AEO emulgel for 1–28 days.

Days	Viscosity (cps)	Spreadability (cm)
0	5769 ± 288.45	6.17 ± 0.3085
7	5765 ± 288.25	6.15 ± 0.3075
14	5754 ± 287.7	5.95 ± 0.2975
21	5752 ± 287.6	5.59 ± 0.2795
28	5700 ± 260	5.29 ± 0.2645

**Table 2:** Comparative MIC determination of the different compounds against *E-coli*.

Sample	MIC (µg/mL)
**Bare AEO**	4.7
**AEO-emulgel**	1.02
**Emulgel**	No

## Data Availability

The data presented in this study are available on request from the corresponding author.
